# Estimated Effect of Climatic Variables on the Transmission of *Plasmodium vivax* Malaria in the Republic of Korea

**DOI:** 10.1289/ehp.1104577

**Published:** 2012-06-18

**Authors:** Young-Min Kim, Jae-Won Park, Hae-Kwan Cheong

**Affiliations:** 1 Department of Social and Preventive Medicine, Sungkyunkwan University School of Medicine, Jangan-gu, Suwon, Republic of Korea; 2 Department of Microbiology, Graduate School of Medicine, Gachon University of Medicine and Science, Yeonsu-gu, Incheon, Republic of Korea

**Keywords:** climate change, climatic variables, distributed lag nonlinear model, generalized linear Poisson model, incubation period, *Plasmodium vivax* malaria, temperate region

## Abstract

Background: Climate change may affect *Plasmodium vivax* malaria transmission in a wide region including both subtropical and temperate areas.

Objectives: We aimed to estimate the effects of climatic variables on the transmission of *P. vivax* in temperate regions.

Methods: We estimated the effects of climatic factors on *P. vivax* malaria transmission using data on weekly numbers of malaria cases for the years 2001–2009 in the Republic of Korea. Generalized linear Poisson models and distributed lag nonlinear models (DLNM) were adopted to estimate the effects of temperature, relative humidity, temperature fluctuation, duration of sunshine, and rainfall on malaria transmission while adjusting for seasonal variation, between-year variation, and other climatic factors.

Results: A 1°C increase in temperature was associated with a 17.7% [95% confidence interval (CI): 16.9, 18.6%] increase in malaria incidence after a 3-week lag, a 10% rise in relative humidity was associated with 40.7% (95% CI: –44.3, –36.9%) decrease in malaria after a 7-week lag, a 1°C increase in the diurnal temperature range was associated with a 24.1% (95% CI: –26.7, –21.4%) decrease in malaria after a 7-week lag, and a 10-hr increase in sunshine per week was associated with a 5.1% (95% CI: –8.4, –1.7%) decrease in malaria after a 2-week lag. The cumulative relative risk for a 10-mm increase in rainfall (≤ 350 mm) on *P. vivax* malaria was 3.61 (95% CI: 1.69, 7.72) based on a DLNM with a 10-week maximum lag.

Conclusions: Our findings suggest that malaria transmission in temperate areas is highly dependent on climate factors. In addition, lagged estimates of the effect of rainfall on malaria are consistent with the time necessary for mosquito development and *P. vivax* incubation.

Climate change is predicted to have a variety of impacts on human health, many of which have been extensively reviewed (Ebi et al. 2006; Mills et al. 2010). Among them, malaria has been recognized as the one of the diseases most sensitive to climate change (Haines et al. 2006; Patz and Olson 2006). Temperature, humidity, and rainfall have been reported to affect the incidence of malaria, either through changes in the duration of mosquito and parasite life cycles or through influences on human or parasite behavior (Paaijmans et al. 2009; Parham and Michael 2010; Snow and Gilles 2002). According to a report by the Intergovernmental Panel on Climate Change (2007), the rate at which temperatures are increasing is higher in the temperate areas of the world.

Although the relationship between malaria and meteorological variables has been assessed in many regions, including Africa, Europe, Asia, South America, and Australia, few studies have been conducted and little is known about the impact of climate variation on malaria in temperate regions (Bi et al. 2003; Zhang et al. 2010). In addition, few relationships between climatic variables other than temperature and *Plasmodium vivax* have been reported. Most studies of meteorological effects on malaria have focused on temperature and rainfall (Parham and Michael 2010; Zhang et al. 2010). Paaijmans et al. (2009) reported that diurnal temperature fluctuation played an important role in parasite development. Moreover, it is known that the longevity of a mosquito increases with increasing relative humidity (Martens et al. 1995) and that mosquito activity decreases with increasing sunshine because mosquitoes are more active during the dark. However, the effects of these variables on malaria have not been estimated. Studies of the effect of various meteorological variables such as the temperature, relative humidity, diurnal temperature range (DTR), the duration of sunshine, and rainfall are needed to clarify the link between malaria and climate and suggest new approaches to reduce the current and future disease burden of malaria.

Since its reemergence in 1993 from the northwest border area facing the Democratic People’s Republic of Korea (DPRK), *P. vivax* malaria has become endemic in the Republic of Korea (ROK) with a peak incidence in 2007 of 2,192 cases, one of the highest among countries in temperate regions (Korea Centers for Disease Control and Prevention 2010; Park 2011). Moreover, the surface air temperature has significantly increased by about 1.5*°*C during the past 100 years on the Korean peninsula (Korea Ministry of Environment 2011), an increase which is greater than the global 0.74*°*C average increase. *P. vivax* malaria continues to be problematic in the northwestern part of the ROK, presenting a seasonal pattern (Park et al. 2009). In 2005 and 2006, the proportion of cases that occurred after a short incubation period increased, suggesting an increase in the length of the transmission period that could be a consequence of rising temperatures in the ROK (Jun et al. 2009; Park 2011; Park et al. 2003). The new emergence and expanding pattern of malaria in the ROK, which has the highest latitude among the temperate region countries, may be evidence of the potential effect of climate change on malaria transmission.

We aimed to estimate the effects of diverse climatic variables, such as temperature, relative humidity, DTR, duration of sunshine, and rainfall, on the transmission of *P. vivax* while taking the lag time into account. We also aimed to provide strategic insights into the current and future impact of climate change on malaria transmission, especially in the temperate regions.

## Methods

*Study area.* During the 1990s, after the initial reemergence of *P. vivax* malaria in the ROK, more than half of the total annual cases were diagnosed among active military personnel and veterans within 24 months of their discharge from military service. However, subsequently, the proportion of civilian cases increased consistently, reaching over 60% in 2006, and the geographical area associated with malaria transmission has expanded southward from the Demilitarized Zone (DMZ), a strip of land running across the Korean Peninsula that serves as a buffer zone between ROK and the DPRK (Jun et al. 2009; Yeom et al. 2005, 2007).

Only civilian cases, which constituted 44–63% of all cases for the years 2001–2009 in the ROK, were included in the analysis. Military cases were excluded because mass chemoprophylaxis has been conducted on a large scale in the military. Thus, *P. vivax* malaria cases among civilians may be more informative for investigating effects of climate on *P. vivax* malaria in the ROK (Jun et al. 2009). We obtained surveillance data for malaria cases, including information about the date of onset and place of residence, from the Korea Centers for Disease Control and Prevention (Osong, ROK), which monitors and manages malaria in the ROK as a nationally notifiable communicable disease. Study cases were restricted to those in the capital region, which covers about 90% of all civilian cases in the ROK and is the only area where malaria is endemic. The capital region includes Seoul, Incheon, and Gyeonggi province and is located in the northwestern ROK, covering 11,730 km^2^ with a combined (census) population of 22,766,850 as of 2005—amounting to over 48% of the entire population of the ROK. Seoul, the capital city of the ROK and the center of its capital area, is located at 37.6*°*N and 127.0*°*E. The study area has a continental climate with four distinct seasons, including a hot and humid summer and a cold and snowy winter.

Daily meteorological parameters, including the daily maximum, mean, and minimum temperature; relative humidity; duration of sunshine; and the amount of rainfall were obtained from eight sites in the capital region that are monitored by the Korea Meteorological Administration (Seoul, ROK). Daily weather data were averaged across the eight sites for use in analyses. Daily DTRs were calculated as the difference between the maximum and minimum temperature on each day and were used as an index of diurnal temperature fluctuation, which can substantially alter the incubation period of malaria parasites and reduce the impact of mean temperature (Paaijmans et al. 2009). Weekly DTRs were calculated as the average of daily DTRs over each week.

Weekly mean values for temperature, relative humidity, DTR, duration of sunshine, rainfall amounts, and numbers of malaria cases were used to estimate the effect of climatic factors on *P. vivax*.

*Statistical analysis.* Generalized linear Poisson regression models allowing for overdispersion were used to examine relationships between the number of malaria cases per week and the climatic variables (McCullagh and Nelder 1989; Zanobetti et al. 2000). We began by using a generalized additive model with natural cubic splines (Hastie and Tibshirani 1990) to characterize the shapes of relationships between *P. vivax* malaria and weather variables while controlling for possible confounders. Models included Fourier terms up to the sixth harmonic per year to account for seasonality in malaria incidence, and indicator variables for each calendar year to account for temporal trends over the study period (Hashizume et al. 2008). After examining the shapes of each exposure–outcome relation to confirm assumptions regarding linearity or identify threshold values, we fitted generalized linear Poisson regression models with natural cubic splines [4 degrees of freedom (df)] to control for confounding factors, including other climatic variables. Final models for each variable of interest (i.e., temperature, relative humidity, DTR, and duration of sunshine) were selected based on model fit using Akaike’s information criterion. We estimated associations between climatic variables and malaria incidence for various single-week lags. For example, a lag of 0 weeks (unlagged) corresponds to the association between weather in a given week and the risk of malaria incidence in that same week. A lag of 8 weeks refers to the association between weather in a given week and malaria incidence 8 weeks later.

The model specifications used for climatic variables except rainfall were as follows:

ln[*E*(*Y*)] = β_0_ + β_1_*X_t_* + Σ*S_i_*(*X_i_*) + sin(2π*t_j_*/N) + cos(2π*t_j_*/N) + *year.*

Here, *E*(*Y*) denotes the number of expected malaria cases; β_1_ is the coefficient (slope) for the weather variable (*X_t_*) during week *t*; *S_i_(X_i_)* denotes the smooth functions for the *i* covariates; *t_j_* denotes the week of the year *j* (*t* = 1, 2*…*52); and N is the period up to the sixth harmonic per year. Weekly mean values for relative humidity and duration of sunshine were included in the model for a 1°C increase in the mean temperature; weekly mean values for temperature, duration of sunshine, and DTR were included in the model for a 10% increase in relative humidity; weekly mean values for duration of sunshine, relative humidity, and mean temperature were included in the model for a 1°C increase in the DTR; and weekly mean values for temperature and relative humidity were included in the model for a 10-hr increase in the weekly duration of sunshine.

To account for a longer and nonlinear lag effect as suggested by Thomson et al. (2006), we examined the relationship between malaria incidence and rainfall by fitting nonlinear unconstrained distributed lag models, a subtype of distributed lag nonlinear model (DLNM) (Armstrong 2006). We adjusted the weekly averaged mean temperature using a cross-basis framework (Gasparrini et al. 2010) to account for the combined effects of a 10-week maximum lag structure (stratified at 4 weeks for mean temperature and polynomial for rainfall) and a nonlinear exposure response represented by using natural cubic splines with 5 df for the effect of rainfall and temperature. The knot for temperature at 4 weeks was included to account for a change in the effect of temperature, which was greater for 2-, 3-, and 4-week lags than for 5- through 10-week lags; the lagged effect of temperature began to decrease after a 4-week lag. After plotting the DLNM for the effect of rainfall without threshold, we estimated the increase in the number of malaria cases for a 10-mm increase in rainfall ≤ 350 mm/week, up to which level the effect of rainfall increased linearly. In addition, we estimated single-day lag effects of an increase in rainfall on the daily (vs. weekly) number of malaria cases to account more accurately for daily variation in rainfall. A generalized linear Poisson regression model with natural cubic splines was utilized for the single-day lag effects of rainfall after examining the shapes of each exposure–outcome relation to confirm assumptions regarding linearity.

All statistical analyses were performed with R software (version 2.13.0; R Project for Statistical Computing, Vienna, Austria) using the packages MGCV (version 1.7–6) and DLNM (version 1.4.0). All tests were two-sided, and an alpha level of < 0.05 was considered significant.

## Results

**Figure 1:**
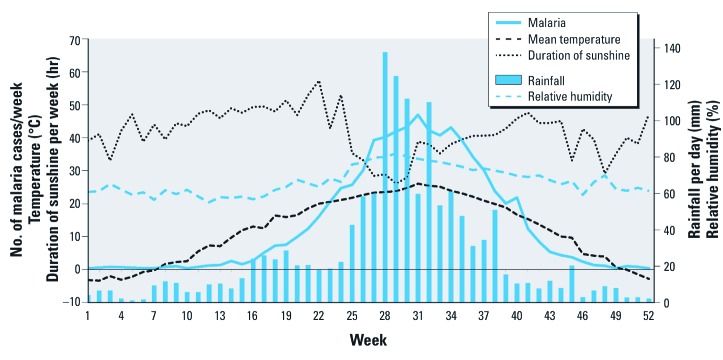
Seasonal variation in the mean weekly number of malaria cases in the capital area and mean weekly climatic variables in the ROK (2001–2009).

Between 2001 and 2009, 6,548 cases of malaria were reported among civilians in the capital area (of 7,557 total cases in the ROK, with an annual maximum of 1,193 cases out of 1,295 total civilian cases in 2007). Over the entire study period (2001–2009), there was a mean (± SD) of 14.0 ± 18.2 malaria cases per week, mean temperature was 12.0 ± 9.9°C, and the mean weekly rainfall was 27.20 ± 49.1 mm (Table 1). A strong seasonal variation in malaria incidence coincided with seasonal variation in mean temperature and rainfall variations (Figure 1), with the highest average number of malaria cases reported during the 31st week.

**Table 1:** Summary of weekly malaria cases and meteorological variables in the capital area of ROK, 2001–2009.

Variable	Mean ± SD	Minimim	Maximum
No. of cases of P. vivax malaria		14.0 ± 18.2		0		89
Mean average temperature (°C)		12.0 ± 9.9		–8.5		27.5
Mean maximum temperature (°C)		17.4 ± 9.8		–3.2		33.2
Mean minimum temperature (°C)		7.3 ± 10.2		–13.7		23.8
Relative humidity (%)		66.8 ± 9.6		40.3		88.5
Mean DTR (°C)		10.1 ± 2.0		4.2		15.8
Rainfall (mm/week)		27.2 ± 49.1		0.0		445.7
Duration of sunshine (hr/week)		41.8 ± 13.3		7.0		79.6

Pearson correlation coefficients between the number of *P. vivax* malaria cases and mean temperature, relative humidity, DTR, duration of sunshine, and rainfall and were 0.74, 0.60, –0.48, –0.22, and 0.44, respectively.

**Figure 2:**
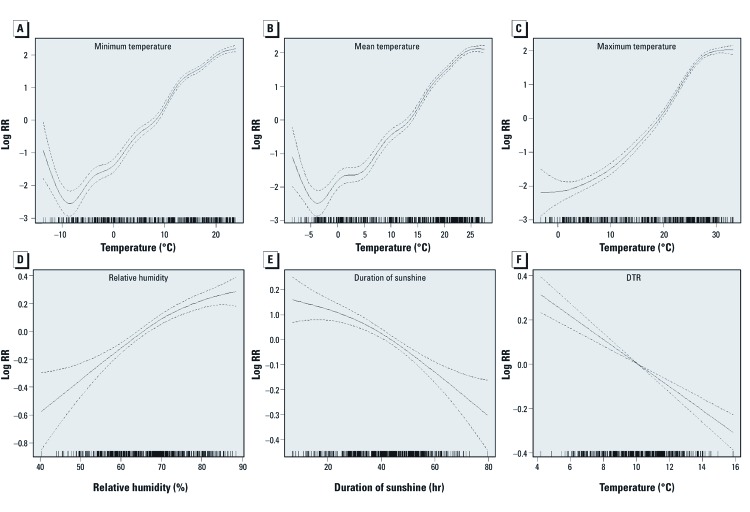
LogRR (solid lines) and 95% CI (dashed lines) for associations between the number of malaria cases per week and temperature [weekly minimum (*A*), mean (*B*), and maximum (*C*)], relative humidity (*D*), duration of sunshine (*E*), and DTR (*F*).

*Estimated effects of temperature, relative humidity, DTR, and duration of sunshine.* Associations between malaria cases and temperature, relative humidity, DTR, and duration of sunshine during the same week estimated using Poisson regression with adjustment for seasonal variation, between-year, and other weather variables are shown in Figure 2. The numbers of malaria cases were positively associated with relative humidity and minimum, mean, and maximum temperature, and negatively associated with DTR and duration of sunshine.

**Figure 3:**
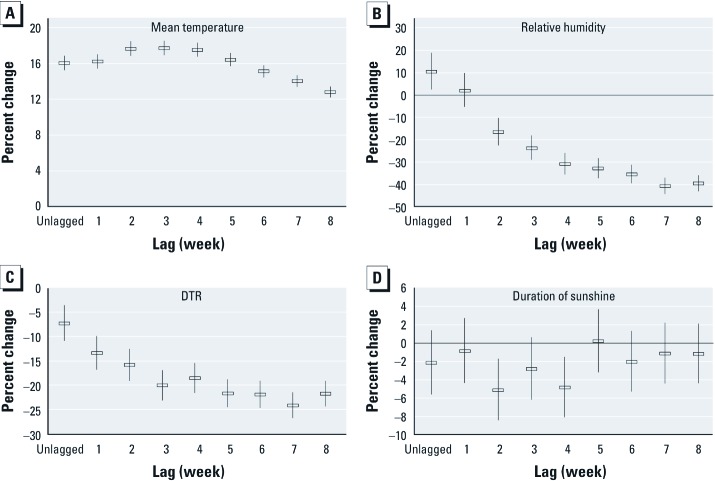
Estimated percent change (95% CI) in the weekly number of malaria cases for a 1°C increase in mean temperature (*A*), 10% increase in relative humidity (*B*), 1°C increase in DTR (*C*), and 10-hr increase in the duration of sunshine (*D*) during the same week (unlagged) and for single-week lags.

Figure 3 shows estimated single-week lag effects of mean temperature, relative humidity, DTR, and duration of sunshine based on generalized linear Poisson regression adjusted for the other climate variables. A 1*°*C increase in mean temperature was associated with a 16.1% [95% confidence interval (CI): 15.3, 16.9%] increase in malaria cases during the same week (unlagged), and with a maximum increase of 17.7% (95% CI: 16.9, 18.6%) after a 3-week lag. A 10% increase in relative humidity was associated with a 10.4% (95% CI: 2.5, 18.9%) increase in malaria cases during the same week. However, numbers of malaria cases decreased in association with a 10% rise in relative humidity during previous weeks with a 40.7% decrease (95% CI: –44.3, –36.9%) when lagged by 7 weeks. A 1°C increase in the DTR was associated with a 7.3% decrease (95% CI: –10.9, –3.5%) in malaria during the same week, and a 24.1% decrease (95% CI: –26.7, –21.4%) when lagged by 7 weeks. A 10-hr increase in the duration of sunshine per week was associated with a 5.1% (95% CI: –8.4, –1.7%) and 4.8% (95% CI: –8.1, –1.5%), decrease in malaria when lagged by 2 and 4 weeks, respectively, after adjusting for mean temperature and relative humidity.

**Figure 4:**
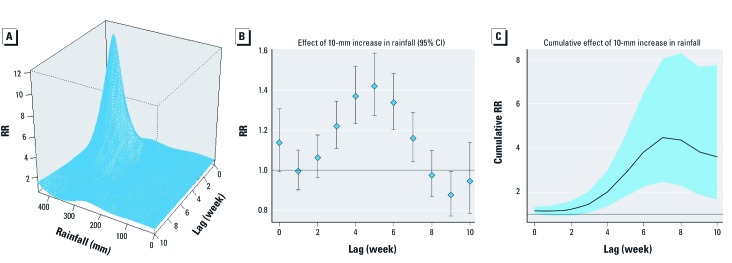
Estimated effect of weekly rainfall on *P. vivax* malaria cases fitted with distributed lagged nonlinear model adjusted for mean temperature, seasonal variation, and between-year variation. (*A*) Three-dimensional image of the associations between increase in weekly rainfall and malaria cases adjusted for temperature with a maximum lag of 10 weeks, (*B*) lag-specific relative risk estimates (95% CI), and (*C*) estimated cumulative lagged RR (95% CI) for a 10-mm increase in weekly rainfall up to 350 mm/week.

*Estimated effect of rainfall.* A three-dimensional plot of the estimated effect of rainfall based on a nonlinear unconstrained distributed lag model with adjustment for the lagged effect of temperature using natural cubic splines (5 df) suggests an effect of weekly rainfall up to a total of approximately 350 mm when lagged 2–4 weeks (Figure 4A). Model estimates, fitted with assumption that the effects of rainfall were absent above 350 mm/week, indicate statistically significant increases in relative risk (RR) for *P. vivax* malaria incidence associated with a 10-mm increase in weekly rainfall after lags of 3–7 weeks (Figure 4B). The cumulative RR for a 10-mm increase in weekly rainfall ≤ 350 mm/week with a 10-week lag is 3.61 (95% CI: 1.69, 7.72) (Figure 4C).

**Figure 5:**
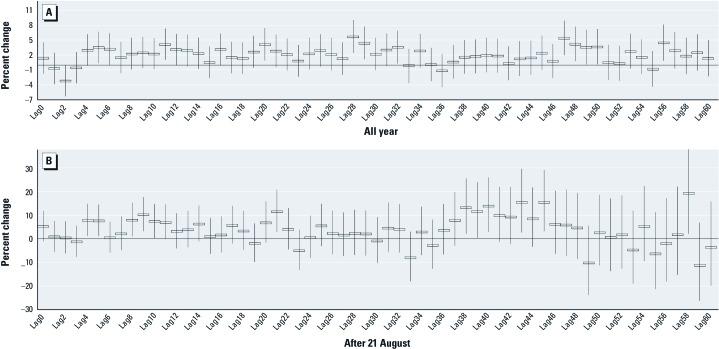
Estimated percent increase (95% CI) in daily malaria incidence expected with a 10-mm increase in rainfall (≤ 50 mm/day) for single-day lags based on all data (*A*) and based on data for 21 August through 31 December only (*B*).

The effects of rainfall on the daily malaria incidence were also estimated in order to determine whether a more specific lag effect is suggested when daily (vs. weekly) data are modeled. Estimated associations based on an adjusted generalized additive model indicated a linear effect ≤ 50 mm/day (data not shown), consistent with an effect of ≤ 350 mm of weekly rainfall as shown in Figure 4A, we estimated the percent change in malaria incidence due to a single-day lag effect of rainfall of < 50 mm/day ≤ 60 days earlier, after adjusting for the daily mean temperature, duration of sunshine, seasonal variation, day of week, and year. Estimated lagged effects were statistically significant for 28-, 47-, 48-, and 56-day lags, with corresponding percent increases in daily malaria incidence with a 10-mm increase in rainfall of 5.7% (95% CI: 2.4, 9.1%), 5.4% (95% CI: 2.0, 8.9%), 4.2% (95% CI: 0.8, 7.7%), and 4.5% (95% CI: 0.9, 8.2%) (Figure 5A).

In addition, we analyzed the effect of daily rainfall after the average peak incidence of malaria during the 31st week of the year (Figure 1, approximately 21 August). Significant lag effects were observed at 38, 40, 43, and 45 days, with the largest percent change at 58 days (19.1% increase with a 10-mm increase in daily rainfall ≤ 50 mm; 95% CI: 2.1, 39.0%) (Figure 5B).

## Discussion

Although *P. vivax* is the prevalent strain of malaria in seasonal climates (i.e., with distinct dry and wet seasons) (Gilbert and Brindle 2009), few studies have been conducted on the association between the transmission of *P. vivax* malaria with climatic variables in temperate areas using empirical and short-interval data. Zhang et al. (2010) estimated effects of climate factors on *P. vivax* malaria in a Chinese city located in a temperate zone using monthly data, but did not include rainfall and humidity in their Seasonal Autoregressive Integrated Moving Average model because they were not statistically significant predictors of malaria incidence. Bi et al. (2003) reported that monthly mean temperature and total monthly rainfall were significant predictors of *P. vivax* malaria after a 1-month lag in Shuchen, China, a subtropical city.

We estimated lagged effects of diverse climatic variables, including temperature, rainfall, relative humidity, DTR, and duration of sunshine, on the incidence of *P. vivax* in a temperate area of the ROK using weekly and daily data, which provided more detailed information on the relationship between climate factors and *P. vivax* malaria than previous studies. We estimated significant effects of weekly rainfall on malaria incidence after accounting for distributed lag effects. Although not directly comparable due to differences in methodology, plasmodium species, and climate zone, our results are consistent with a study of *Plasmodium falciparum* in the East African highland that reported a 6–138% increase in malaria incidence with a 22% increase in monthly rainfall (Zhou et al. 2004).

The main advantage of the distributed lag model is that it can incorporate a detailed representation of the time-course of the exposure–response relationship, and thereby estimate the overall effects of climate variables in the presence of lagged effects or harvesting. DLNM is an extension of the distributed lag model that includes functions describing the shape of the relationship between exposure and response and its distributed lag effects (Gasparrini et al. 2010) simultaneously. We estimated the relation between rainfall and malaria incidence adjusted for confounding by temperature, which was modeled using natural cubic splines (5 df) and a moving average with ≤ 10 weeks of lag with a 4-week lag knot to reflect the change in the estimated effect of the mean temperature on malaria at 4 weeks. When the temperature was modeled using a polynomial lag type instead of the moving average, the estimated relative risk for the cumulative effect of rainfall increased from 3.61 (95% CI: 1.69, 7.72) to 5.02 (95% CI: 2.13, 11.8). However, when we adjusted for the duration of sunshine per week in addition to temperature (modeled as a moving average for lagged effect), the cumulative effect of rainfall decreased to 2.95 (95% CI: 1.19, 7.31). When the maximum lag was prolonged to 12 weeks, the overall cumulative effect of rainfall was no longer significant (RR 1.98; 95% CI: 0.76, 5.22), whereas the RR increased to 4.19 (95% CI: 2.30, 7.65) with a maximum lag of 8 weeks after adjusting for temperature.

In temperate regions, the onset of primary symptoms after *P. vivax* infection reflects two different incubation periods: short and long (or intermediate). If the sporozoite injected by a mosquito into a human host develops directly into a tissue schizont, the incubation period between the initial infection and the onset of symptoms is short, typically from 10 days to 4 weeks, resulting in an early primary attack. Conversely, if the sporozoite develops into a dormant hypnozoite (rather than a tissue schizont) in liver cells, the onset of illness may be delayed for ≤ 1 year, resulting in a long incubation period and late primary attack (Hankey et al. 1953; Sinden and Gilles 2002). Nishiura et al. (2007) estimated that the average length of a short incubation in the ROK is 26.6 days (95% CI: 21.0, 32.2 days).

In addition to the incubation period between infection and symptoms, the association between rainfall and malaria incidence also reflects the time required for mosquito larvae to develop into adult mosquitos, and the time required for *P. vivax* gametocytes to develop into infectious sporozoites after an adult mosquito has taken a blood meal from an infected human host [referred to as the extrinsic incubation period (EIP)]. We estimate that the EIP for *P. vivax* in the ROK is approximately 12–14 days according to the formula DD/(T – Tmin), where degree-days (DD) represent the required accumulation of temperature units over time (estimated to be 105 DD for *P. vivax*), Tmin indicates the minimum temperature for parasite development (14.5–15°C for *P. vivax*) (Martens et al. 1995), and T indicates the average temperature (23°C during 2001–2009). In addition, the estimated time for the development of an adult mosquito from an egg to adult at 22–26°C has been reported to be 11.2–17.6 days (Bayoh and Lindsay 2003). Therefore, the time required to complete mosquito development and the EIP would delay the apparent effect of increased rainfall on malaria incidence by at least 23–32 days.

Based on the estimated time required for mosquito development, the EIP, and a short incubation period before the onset of symptoms, we would expect the effect of rainfall on malaria incidence to be lagged by approximately 42–61 days. Models based on daily data after 21 August, the approximate peak in annual malaria incidence during the study period, indicated clear lag effects at 43–45 days, with the largest increase at 58 days. These estimated effects were much greater than estimates based on data for the entire year, which is consistent with our hypothesis that the majority of malaria cases occurring after late August represented a primary attack after a short incubation period. In contrast, we hypothesize that incidences at other times during the year would include a larger proportion of late primary attack after a long incubation that would have a much weaker temporal relation (if any) with rainfall and other climatic variables.

Parham and Michael (2010) reported that the malaria transmission rate strongly depends on the vector (mosquito) density, and that changes in rainfall govern malaria endemicity, invasion, and extinction by influencing mosquito abundance. Although it has been assumed that effects of rainfall would be less predictable and more difficult to quantify than effects of temperature (Bi et al. 2003; Zhang et al. 2010), our analyses suggest a clear relationship between rainfall and malaria transmission in temperate regions with a lag effect consistent with the time required for the development of the mosquito, the EIP, and the incubation period in the human body.

The estimated effect of a 1°C rise in weekly mean temperature on the incidence of *P. vivax* malaria in the ROK (17.7% increase; 95% CI: 16.9, 18.6%) was larger than the 11.8–15.8% increase estimated for the city of Jinan, China, which is also located in a temperate region (Zhang et al. 2010). Increase in relative humidity had a positive effect on malaria incidences during the same week. However, numbers of malaria cases decreased in association with a rise in relative humidity when lagged by 2–8 weeks. We estimated a negative effect of DTR on malaria with a long lag period in accordance with previous research by Paaijmans et al. (2009) who reported that large temperature fluctuations can slow increases in malaria under warm conditions; effects that will tend to lessen the impact of increases in mean temperature. Our results also showed that increase in duration of sunshine was associated with decrease in malaria, which is reasonable when considered that the main hour of activity of mosquitoes is after sunset. The present study provides strategic insights into the current and future impact of climate change on malaria transmission, especially for *P. vivax* malaria in temperate regions, based on reliable daily and weekly data on malaria incidence, which must be reported when diagnosed in the ROK.

## Conclusions

The incidence of malarial infection at a relatively high latitude area appears to depend strongly on humidity, DTR, duration of sunshine, and rainfall as well as the temperature. Lagged effects estimated for the association between rainfall and malaria are consistent with expectations given the time necessary for mosquito development, *P. vivax* development, and the onset of malaria symptoms. Effects of climate change on rainfall, temperature and other climatic variables may increase the range of populations at risk of *P. vivax* infection, especially in temperate regions.
